# Bridging two hosts: how intracellular environments shape flaviviral infection

**DOI:** 10.1128/mbio.01305-26

**Published:** 2026-06-23

**Authors:** Pavithra Umashankar, Shwetha Shivaprasad

**Affiliations:** 1 Molecular Biology and Genetics Unit, Jawaharlal Nehru Centre for Advanced Scientific Research 29130, Bangalore, India; Albert Einstein College of Medicine, Bronx, New York, USA

**Keywords:** flavivirus, mosquito, host-virus interactions, stress response

## Abstract

Mosquito-borne flaviviruses replicate in physiologically and biochemically distinct host environments in humans and mosquitoes, providing a unique window into conserved and host-specific mechanisms shaping viral infection efficiencies and outcomes. This review focuses specifically on intracellular factors, including proteins, metabolites, innate immune effectors, and stress sensors in human and mosquito cells that collectively regulate the flaviviral life cycle and host cell survival, with specific emphasis on dengue virus. We discuss both conserved dependencies and species-specific differences in receptor usage, membrane remodeling, RNA translation, and replication strategies that influence viral dynamics across hosts. We further highlight how host metabolism, innate immune sensing, and stress response pathways drive divergent outcomes in virus-infected cells. In mammalian cells, rapid viral replication activates interferon-mediated antiviral responses that limit viral infection, but also lead to cytopathic effects and apoptosis. In contrast, mosquito cells support persistent, non-cytopathic infection mediated by RNA interference-dependent control of viral replication, coupled with antioxidant and anti-apoptotic defenses that maintain cellular homeostasis. This comparative perspective integrates insights from mammalian and mosquito systems to illustrate how host environments shape flaviviral infection, host susceptibility, and infection outcomes. Identifying these intracellular determinants of infection and persistence will be critical for defining host susceptibility, understanding barriers to cross-species transmission, and predicting viral emergence potential.

## INTRODUCTION

Flaviviruses are single-stranded, positive-sense RNA viruses transmitted by arthropods such as mosquitoes and ticks to a broad range of vertebrate hosts. Mosquito-borne flaviviruses collectively infect ~400–500 million people annually and include major human pathogens such as dengue virus (DENV), Zika virus (ZIKV), West Nile virus (WNV), and Japanese encephalitis virus (JEV) (1, 2). DENV alone accounts for more than 65% of these infections. Rapid urbanization, climate change, and increased global travel have facilitated the expansion of *Aedes* and *Culex* mosquitoes into previously non-endemic regions, driving the global emergence of these viruses (3–5). The lack of broadly effective antiviral therapeutics and limited efficacy of available vaccines highlights an urgent need for a deeper mechanistic understanding of how these viruses establish infection, adapt to divergent host environments, and drive pathogenesis, enabling the design of more effective interventions.

The ability of mosquito-borne viruses to alternate between human and mosquito hosts makes them ideal candidates to study both conserved and species-specific determinants of viral infection. Phylogenetic evidence suggests that flaviviruses originated as insect-specific viruses and co-evolved with their mosquito vectors over long evolutionary timescales, gradually acquiring the ability to infect vertebrate hosts (6, 7). As a result, these viruses have evolved strategies to replicate efficiently, evade immune sensing, and establish persistent, largely non-cytopathic infections in mosquitoes, enabling repeated transmission to vertebrate hosts throughout the lifespan of the mosquito. In contrast, infections in humans are acute and require rapid viral replication to achieve sufficiently high viremia for transmission during a mosquito blood meal. The constraints of transmission therefore impose fundamentally different demands on viral replication dynamics in each host: slow, persistent infection in the mosquito versus rapid systemic amplification in humans (8). In addition, the innate and adaptive immune responses that contribute to viral clearance in humans can also drive immune dysregulation, inflammation, and endothelial dysfunction, leading to severe disease outcomes (9, 10).

These differences in infection dynamics and outcomes across hosts arise due to a combination of environmental factors (such as temperature, pH, nutrient availability, and microbiota), extracellular barriers (including physical and anatomical defenses as well as systemic immune regulators), and intracellular determinants (such as host protein availability, organelle dynamics, metabolic state, and innate immune signaling). In this review, we focus on the intracellular determinants that regulate flaviviral infection in human and mosquito cells, with a specific focus on DENV, which has been the primary focus of most studies in this area. We highlight key similarities and differences in host-virus interactions across these systems and identify critical gaps in our understanding of conserved and species-specific host factors that regulate viral replication, cytopathic effects, and persistence. Defining the molecular and cellular constraints that regulate infection in each host will provide a mechanistic basis for designing targeted antiviral interventions, improving infection outcomes, and predicting which viruses are likely to cross species barriers.

### DENV life cycle

DENV enters host cells through interactions of the viral envelope (E) protein with a broad range of cell surface attachment factors, including glycosaminoglycans and C-type lectins, followed by receptor-mediated endocytosis (11, 12). Acidification of the endosomal lumen triggers conformational rearrangements in the E protein, which exposes the fusion peptide and mediates fusion of the viral envelope with the endosomal membrane to release the encapsidated viral RNA into the cytoplasm (13–15). The DENV genome is a single-stranded, positive-sense ~11 kb RNA consisting of a single open reading frame (ORF) and flanked by highly structured 5′ and 3′ untranslated regions (UTRs) (16, 17). The cap structure at the 5′ end of the viral RNA is recognized by the eIF4F cap-binding complex to initiate translation of the viral polyprotein, which is co- and post-translationally processed into three structural (C, prM, E) and seven non-structural (NS1–NS5) proteins (18). NS5, the viral RNA-dependent RNA polymerase, coordinates the switch from translation to replication and catalyzes viral RNA synthesis (19, 20). Both viral RNA translation and replication take place within ER-derived double-membraned vesicles, which shield the virus from host cellular nucleases and proteases (21). Newly synthesized positive-sense genomes exit the double-membraned replication compartments and associate with viral capsid proteins to form nucleocapsids. The DENV NS2A protein plays a key role in coordinating virion assembly by simultaneously interacting with the 3′ UTR of the viral RNA, the structural proteins (C-prM-E), and the NS2B-NS3 protease complex (22). Proteolytic cleavage of the C-prM-E polyprotein by the NS2B-NS3 protease and host signal peptidases allows capsid dimerization and subsequent interactions with prM-E heterotrimers to form immature virions. prM is then cleaved by host furin to generate mature, infectious virions that are released from the cells by exocytosis. Efficient completion of the viral life cycle in infected cells depends on the availability of host factors, the cellular metabolic state, and innate and stress response pathways (23–25), which together regulate the efficiency of virion production and the extent of cellular damage. These intracellular determinants and the mechanisms by which they influence infection are discussed in detail in the following sections.

### Host factor availability

**Fig 1:**
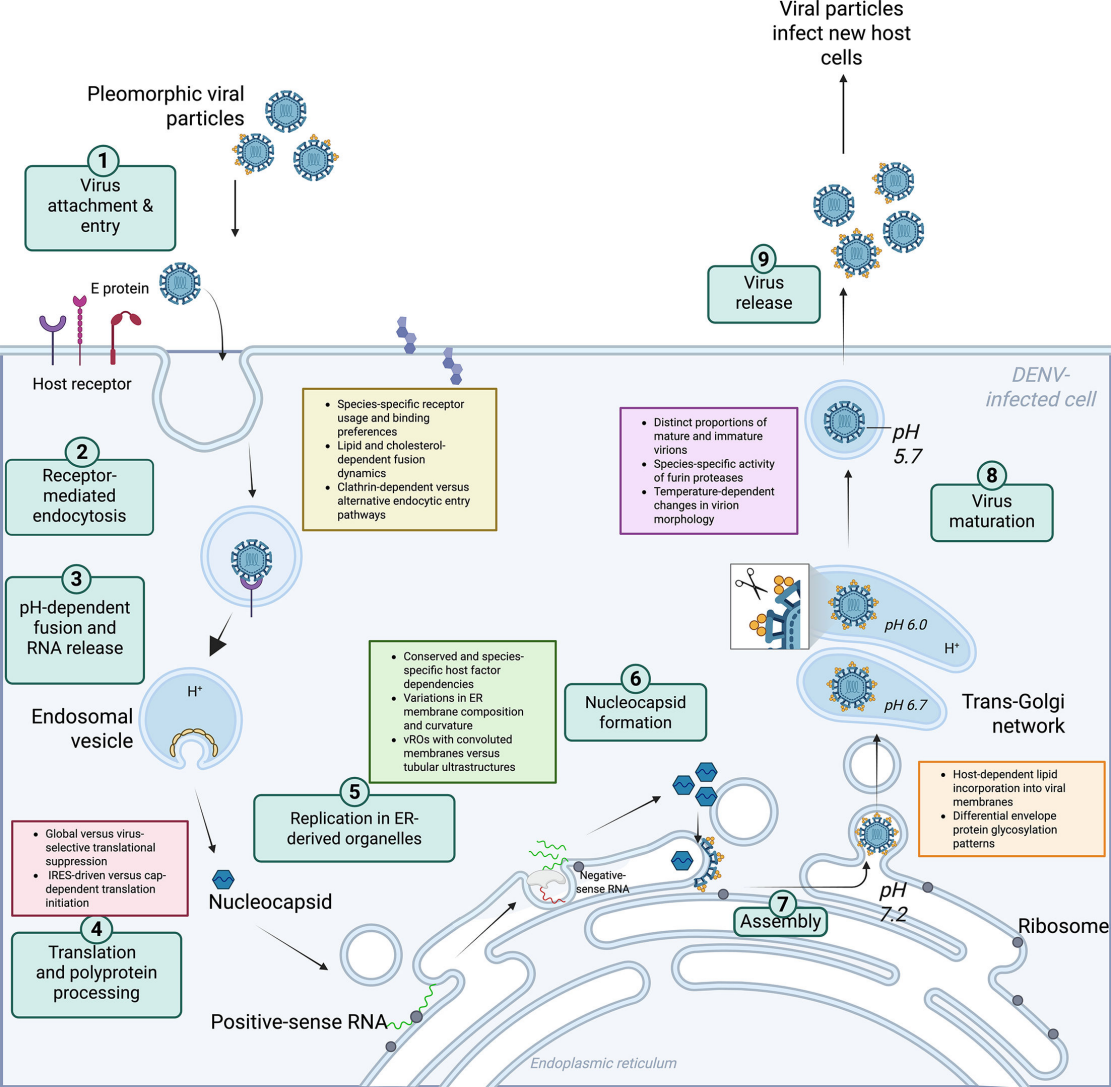
Host-specific determinants regulating the flaviviral life cycle. Schematic overview of the flaviviral life cycle highlighting key molecular and cellular differences that influence infection in mammalian and mosquito hosts. Viral entry is shaped by species-specific receptor usage, lipid- and cholesterol-dependent membrane fusion dynamics, and the utilization of clathrin-dependent or alternative endocytic pathways. Following fusion and vRNA release into the cytoplasm, translation is initiated through canonical/non-canonical cap-dependent or internal ribosome entry site (IRES)-like mechanisms. High levels of viral protein synthesis impose significant stress on the endoplasmic reticulum (ER), activating ER stress response pathways that can trigger either global translational shutdown or selectively repress viral RNA translation depending on the host. Viral replication and virion assembly occur in close association with ER-derived membranes and are influenced by host factor availability, ER membrane composition and curvature, and the formation of distinct membrane ultrastructures. During maturation within the trans-Golgi network, differences in furin cleavage efficiency and temperature-dependent structural transitions contribute to the generation of structurally distinct, often pleomorphic, viral particles. Host-dependent variations in virion lipid composition and envelope glycosylation further influence viral infectivity and transmission across hosts. (Created in BioRender [S. Shivaprasad, 2026, https://BioRender.com/x7vx3i6].)

The availability of host factors, including cellular proteins, RNAs, intracellular membranes, and metabolites, has a significant impact on each stage of the viral life cycle. While host factors co-opted by DENV and other flaviviruses across both human and mosquito cells are largely conserved due to the overall similarities in the viral life cycle, a few species-specific differences are also observed that modulate host susceptibility and viral replication efficiencies (Fig. 1).

#### Receptor usage and viral entry

DENV, like most flaviviruses, encodes a single envelope glycoprotein that binds to a wide range of cell surface receptors, including glycosaminoglycans, glycosphingolipids, C-type lectins, laminins, and heat shock proteins across both human and mosquito hosts to mediate viral entry (12, 26). However, receptor preferences may vary when specific receptors are absent in one host or when amino acid differences in receptor orthologs prevent the viral envelope from binding with equal efficiency across hosts. For instance, TIM-1 is a cell surface receptor that mediates ZIKV entry into mammalian cells. But mosquito cells lack TIM-1 orthologs, and mutations in the viral envelope protein that disrupt TIM-1 binding do not affect viral entry into mosquito cells, indicating that the virus utilizes alternative receptors for entry into mosquito cells (27). Despite the identification of several entry receptors in mammalian systems through unbiased genetic screens and surface proteomics, comparable studies in mosquito cells are still lacking, limiting our understanding of the full repertoire of receptors that support viral entry in this host. Recently, deep mutational scanning has emerged as a powerful tool to map interactions between viral envelope proteins and host receptors. Although such studies have not yet been applied to flaviviruses, work with Chikungunya virus (CHIKV), an alphavirus, has identified mutations in the viral envelope that differentially affect entry into mosquito versus human cells, suggesting that certain interactions can be more important for entry in one host cell type rather than the other (28). In addition to viral envelope protein sequence, the type of glycans that are expressed on the viral envelope can also impact receptor usage. Studies with WNV and alphaviruses have shown that mosquito-derived virions incorporate high-mannose glycans with exposed terminal mannose residues on their envelopes, allowing them to bind more efficiently to C-type lectin receptors such as DC-SIGN. In contrast, virions produced by mammalian cells have relatively complex glycans that bind these receptors less efficiently (29, 30).

Even across mosquito genera, it is observed that DENV replicates preferentially in the midguts of *Aedes* but not *Culex* mosquitoes, which may reflect differences in the ability of the virus to enter midgut epithelial cells. For example, the DENV E protein interacts with a ~35 kDa protein present in the midgut brush border membrane fractions (BBMFs) of *Aedes* mosquitoes, but this receptor seems to be absent from *Culex* BBMFs (31). Similarly, the Western equine encephalitis virus (WEEV) envelope binds strongly to BBMFs derived from WEEV-susceptible *Culex* strains, but not to BBMFs derived from WEEV-refractory *Culex* strains, suggesting that differences in the expression or binding affinities of midgut surface proteins can act as barriers to viral entry and midgut infection (32). Thus, the availability and utilization of cell surface receptors across human and mosquito hosts, as well as between susceptible and refractory mosquito species, can vary widely and warrant further studies.

Viral receptors and attachment factors are often concentrated in lipid rafts and cholesterol-rich plasma membrane microdomains on the cell surface, which promotes interactions of the viral envelope glycoprotein with cell membranes and subsequent clathrin-mediated endocytosis (CME) of virions. The lipid composition of host and viral membranes, including the presence of cholesterol, sphingolipids, and negatively charged phospholipids, significantly impacts membrane fluidity and viral envelope-receptor interactions (33, 34). Given that mosquitoes are cholesterol auxotrophs and contain almost 80% lower cholesterol amounts in their membranes compared to mammalian cells (35), differences in the kinetics/efficiency of membrane fusion or a requirement for additional accessory lipids/proteins may be expected. While the adaptor and scaffold proteins required for CME are highly conserved across phyla, flaviviruses also utilize macropinocytosis and clathrin-independent dynamin-dependent pathways for entry into mammalian cells, depending on the viral serotype and host cell type being studied (36–39). It is not known whether these alternative routes are also employed for viral entry into the mosquito midgut or salivary glands. Biophysical methods such as single-particle tracking, live-cell imaging, and cryo-electron microscopy combined with spatial lipidomics studies can reveal how viruses reorganize host membrane lipids during attachment, fusion, and entry across different host cells and identify novel targets for blocking viral entry.

#### Translation initiation strategies

A distinguishing feature of orthoflaviviruses is the presence of a type I cap structure (m⁷GpppN) at the 5′ end of their genomic RNA. The cap structure is recognized by the eIF4F cap-binding complex, consisting of eIF4E (cap-binding protein), eIF4G (adaptor protein), and eIF4A (helicase). This is followed by recruitment of the 40S and 60S ribosomal subunits to the viral RNA to initiate cap-dependent translation and synthesis of viral structural and non-structural proteins. Often, flaviviral RNAs have adenosine as the first nucleotide after the cap, which plays a regulatory role in eIF4E binding and function (40). However, for some flaviviruses like DENV, viral translation remains largely unaffected by depletion of eIF4E, suggesting that the virus can bypass the requirement for a functional m⁷G cap under stress (41). When DENV-infected cells shut down global translation to counter viral infection, the viral RNA continues to be actively translated, suggesting that cap-independent translation may be essential at these later stages of infection for sustained viral protein synthesis (25). Two distinct non-canonical modes of translation initiation have been described for DENV in mammalian cells. The first one involves an eIF4E-independent, cap-dependent mechanism of translation where viral RNA is translated under limiting eIF4E levels, likely facilitated by alternative initiation factors such as DAP5, eIF3d, or eIF4E2. Long-range interactions between the 5′ and 3′ untranslated regions of the viral genome are thought to play an important role in this process (41, 42). The second pathway is a cap-independent internal ribosome entry site (IRES)-dependent mechanism. Both DENV and ZIKV 5′ UTRs have been shown to contain an IRES that is active when canonical translation initiation is suppressed (43, 44). More recently, ZIKV RNA has been shown to harbor multiple upstream ORFs that enable efficient translation under stress conditions (45). Surprisingly, mosquito cells do not support DENV IRES-driven translation when tested using dicistronic constructs, indicating that trans-acting factors required for efficient IRES activity may not be available in these cells (43, 44, 46). Furthermore, global shutdown of host translation is not well established in infected mosquito cells, so the necessity for alternative translation initiation mechanisms and their functional relevance in this host remains an open question. Supporting this hypothesis, recent studies with ZIKV and CHIKV have shown that host translation remains largely unaffected in infected mosquito cells, and alternative strategies that promote viral translation in human cells, such as remodeling of the tRNA epitranscriptome, are not engaged in mosquito cells (47, 48). These studies also showed that viral translation is selectively repressed over time in persistently infected mosquito cells, suggesting that mosquito cells may employ distinct translation regulatory pathways that differentially modulate viral protein synthesis during infection.

#### Membrane remodeling and replication dynamics

A hallmark of DENV infection is the rearrangement of ER membranes to form viral replication organelles (vROs) (21, 49). vROs comprise (i) vesicle packets, which are believed to be the site of viral RNA replication; (ii) virus bags, which are ER cisternae in which immature, assembled virions accumulate; and (iii) convoluted membranes (CMs), which are bundled, smooth ER membranes enriched in NS3, NS4B, and NS4A (50–53). Recent studies showed that CM biogenesis is positively correlated with mitochondrial elongation and disrupts ER-mitochondrial contact sites, impairing RIG-I-mediated antiviral signaling and thereby facilitating DENV replication in mammalian cells (54). Interestingly, electron microscopy and tomography studies suggest that CMs are absent in DENV-infected mosquito C6/36 cells (55). Since mosquitoes lack a canonical interferon (IFN) signaling pathway, the role of CMs in disrupting ER-mitochondrial contact sites and suppressing RIG-I signaling, as seen in mammalian cells, may not be relevant in mosquito hosts. Also, since C6/36 cells are Dicer-deficient and support abnormally high levels of viral replication leading to apoptosis of infected cells (56), the kind of membrane ultra-structures observed in this cell line may not be similar to those seen in immune-competent mosquito cells, which support low-level, persistent viral infection. In fact, tick cells that are persistently infected with another flavivirus, Langat virus, show more abundant and marked changes in ER structure, including the formation of a large number of tubular structures, which are not seen in acutely infected cells (57). Investigating these differences in mosquito cells that are acutely or persistently infected with single-host and dual-host flaviviruses will provide important insights into their functional roles in viral infection.

Genetic and proteomic screens have identified numerous host factors that are essential for efficient membrane remodeling and viral replication in mammalian cells (58–61), including ER-associated proteins of the oligosaccharyltransferase (OST) complex, the translocon-associated protein complex, and the ER-associated degradation pathway, as well as RNA-binding proteins and immune effectors that interact with viral RNA or viral proteins (62–65). Many of these host requirements are conserved between human and mosquito cells, given the overall similarity of the DENV replication cycle (66, 67). Comparative host-virus protein interactome studies with DENV, ZIKV, and YFV have revealed shared cellular modules exploited by these viruses in both human and mosquito hosts (63, 68), thereby reducing the need for host-specific adaptations that could compromise fitness in either host. Nevertheless, host-specific differences in host factor engagement do exist, likely due to variations in protein sequence, structure, and function. For example, components of the OST complex, which are essential for DENV replication in human cells, are dispensable in mosquito cells. Silencing OST subunits or treatment with the OST inhibitor NGI-1 effectively blocks viral replication in human cells but not in mosquito cells (69, 70). Identifying such differential dependencies will require integrated functional genomics and proteomics approaches, including comparative CRISPR screens, cross-species complementation experiments, and interactome mapping, in both human and mosquito cell systems, to uncover species-specific barriers to infection.

Host factor availability can also impact DENV replication across different mosquito species. For instance, DENV titers in *Culex quinquefasciatus* midguts are significantly lower compared to the *Aedes aegypti* midguts (71). Similarly, ZIKV replication is also severely impaired in the midguts of *Culex* mosquitoes when compared to *Aedes*, despite intrathoracic injection of the virus, suggesting the presence of a strong midgut infection barrier (72). These differences may arise from variation in host factor availability or structural and functional divergence in proteins that are essential for the viral life cycle. An excellent example supporting this idea comes from recent work by Gong Cheng and colleagues, who demonstrated that subtle sequence differences in the mosquito valosin-containing protein (VCP) between *Aedes* and *Culex* mosquitoes can determine their ability to support distinct flaviviruses. They identified two amino acid residues in *Aedes* VCP (D723/N728) that enable specific interaction with the DENV capsid, whereas the corresponding residues in *Culex* VCP (E723/E728) can only interact with the JEV capsid. Importantly, ectopic expression of *Aedes* VCP in *Culex* cells rendered them susceptible to DENV, as it could now bind to the DENV capsid and facilitate packaging of viral nucleocapsids into protective extracellular vesicles for dissemination (73). Such cross-species complementation experiments are critical for uncovering host-specific dependencies that influence vector competence and viral transmission.

#### Assembly, maturation, and virion release

Flaviviral particles are assembled into virions at the ER membranes adjacent to vROs, where coordinated interactions between viral proteins, host factors, and membrane lipids drive particle formation and budding. Host factors such as BiP, calnexin, and protein disulfide isomerases facilitate virion assembly by ensuring the proper folding and maturation of the viral prM and E proteins (49). The lipid composition of the ER membrane, particularly the presence of cholesterol, phospholipids, and sphingolipids, regulates membrane curvature and efficiency of virus budding. Consequently, differences in membrane lipid composition between human and mosquito cells, including variations in cholesterol levels and phospholipid species, can significantly influence virion assembly and production across hosts (74).

As the immature virions transit through the Golgi, the prM protein is cleaved by host furin to form mature virions (75). While the overall steps involved in viral assembly and maturation are largely conserved between human and mosquito cells, host-specific factors strongly influence both prM cleavage efficiency and the structural conformation of released virions. Notably, the efficiency of prM cleavage is significantly lower in mosquito cells compared with mammalian cells. Over 40% of the virions released from infected C6/36 cells are immature or partially mature and show reduced infectivity (76). Although the mechanisms underlying differences in prM cleavage are not fully understood, variations in expression levels and substrate specificity of host furin and furin-like proteases in the trans-Golgi network can influence prM processing. Supporting this idea, studies on Sindbis virus have shown that mammalian and insect furin proteases differentially process the viral envelope protein depending on the amino acid present at the +1 position of the canonical cleavage site. Envelope proteins containing valine or leucine at this position are poorly processed by vertebrate furin but are efficiently cleaved by insect furin, highlighting how host-specific protease preferences influence virion maturation (77). In addition to proteolytic processing, host glycosylation pathways also modify viral envelope proteins in a host-dependent manner, as discussed in previous sections (78). Insect cells predominantly generate virions containing high-mannose glycans on their surface, whereas mammalian cells produce virions with more complex glycan structures, likely due to the absence or lower expression of enzymes essential for the synthesis of complex glycans in insect cells. These differences significantly impact virion stability and receptor interactions during subsequent infections, as discussed earlier.

Temperature also plays an important role in determining the conformation of assembled virions, envelope protein mutations, and rearrangements. At human host temperature (37°C), assembled virions adopt an expanded, bumpy morphology that enhances infectivity in human cells, likely by mimicking a fusion intermediate important for cell entry. In contrast, at mosquito vector temperatures (~25°C), virions predominantly adopt a compact, smooth morphology, although both forms can coexist (79, 80). A detailed discussion of the broader impacts of temperature on the viral life cycle and host survival is covered elsewhere (81).

### Host metabolic environments

Since viral replication and protein synthesis are highly energy-intensive processes, host metabolites, including carbohydrates, lipids, and nucleotide pools, serve as key sources of energy and raw materials. Flaviviruses extensively reprogram host metabolic pathways, including cholesterol and lipid biosynthesis pathways, to directly support viral replication processes and, more broadly, to establish a host cellular state favorable for infection, persistence, and transmission.

Flaviviruses are highly dependent on host cholesterol homeostasis for productive infection. In addition to viral entry, cholesterol and raft-associated lipids are also an important component of viral replication organelles and support viral replication in both mammalian and mosquito cells (82, 83). However, mosquitoes face unique constraints since they cannot synthesize cholesterol *de novo* and rely entirely on dietary sources. Approximately 80%–90% of cellular cholesterol in mosquito Aag2 cells exists in an unesterified form and is stored in the perinuclear compartment where viral replication organelles assemble. The sterol carrier protein 2 (SCP-2) plays an important role in this process, as it helps in transferring cholesterol from other parts of the cell to the perinuclear membranes. Inhibition of SCP-2, either pharmacologically using the inhibitor SCPI-1 or by RNA interference (RNAi), significantly reduces DENV titers in mosquito cells, correlating with depletion of perinuclear cholesterol pools (84). In contrast, SCP-2 is dispensable for DENV replication in mammalian cells. While >90% of SCP-2 is cytosolic in mosquito cells, the protein is localized primarily to peroxisomes, ER, and mitochondria in mammalian cells, highlighting a host-specific dependence of DENV on cholesterol trafficking pathways.

In addition to cholesterol homeostasis, DENV infection induces substantial remodeling of host lipid biosynthetic pathways. The abundances of fatty acids, acyl-carnitines, and sphingolipids are seen to be higher in DENV-infected cells in both human and mosquito hosts (85–88). Interestingly, ceramides, which are precursors for sphingolipid biosynthesis, have contrasting effects on viral replication in the two hosts (85, 86). Ceramides inhibit DENV replication in mammalian cells, whereas in mosquito Aag2 cells, ceramides facilitate viral replication, resulting in increased viral titers, but the mechanistic basis for this remains to be studied (85). Beyond their role in replication, ceramides are also key components of extracellular vesicles that contribute to virion dissemination in mosquitoes and may further influence viral transmission dynamics (89, 90). In the context of viral transmission, recent lipidomic studies in DENV-infected mosquitoes have demonstrated that phospholipid remodeling is essential for DENV replication in infected mosquito cells, and the presence of phospholipid precursors in human blood significantly reduces viral replication in mosquitoes, inhibiting human-to-mosquito transmission (91). Conversely, another class of lipids, sphingomyelins, are highly enriched in mosquito saliva and enhance flaviviral infection in skin cells during a mosquito bite, promoting mosquito-to-human transmission (92). Together, these findings highlight how metabolite compositions in host blood and vector saliva may function as competing biochemical strategies to influence viral transmission dynamics.

Infection-induced remodeling of host lipid metabolism is also reflected in the composition of progeny virions. Lipidomic analyses of DENV virions produced in mammalian and mosquito cells have further revealed limited overlap in virion lipid composition between the two hosts and significant differences in the abundance of key phospholipids, such as phosphatidylethanolamine and phosphatidylserine (74). These differences are not fully explained by infection-induced changes in cellular lipid abundances, suggesting there are other ways by which lipids are selectively incorporated into virions.

Beyond lipid remodeling, flaviviral infection induces substantial changes in central carbon metabolism and cellular energy utilization to favor anabolic pathways that supply ATP and biosynthetic precursors necessary for efficient viral replication. In DENV-infected mammalian cells, glycolysis is significantly upregulated, with increased expression of both glucose transporter 1 and hexokinase 2, which promote glucose uptake and glycolytic flux (93, 94). Additionally, mitochondrial β-oxidation and oxidative phosphorylation are also enhanced to meet the high ATP requirements (95). Similarly, in mosquito cells, DENV infection enhances glucose production from trehalose, which is the major circulating sugar (96). In addition to being fully oxidized for ATP production, glucose-derived carbon intermediates may also be diverted toward fatty acid biosynthesis, supporting membrane biogenesis and viral replication in both hosts (97, 98). Interestingly, studies with ZIKV have revealed that glucose is metabolized through distinct biochemical pathways in human and mosquito hosts, resulting in divergent outcomes. In mammalian cells, ZIKV infection promotes glucose utilization through the TCA cycle, leading to altered AMP/ATP ratios, AMPK activation, and subsequent caspase-mediated cell death. In contrast, in mosquito cells, glucose is preferentially directed through the pentose phosphate pathway without substantially perturbing ADP/ATP ratios, thereby maintaining energy homeostasis and promoting cell survival (99). These findings highlight that while flaviviruses exploit glucose metabolism in both mammalian and mosquito hosts, the underlying metabolic routes and cellular outcomes may be different.

Overall, the host metabolic landscape is extensively reprogrammed during flaviviral infections to meet the biosynthetic demands of viral replication and to regulate viral transmission efficiencies. Metabolites are often differentially distributed or redirected through distinct biochemical pathways in different hosts, which can influence infection outcomes. Understanding these host-specific metabolic adaptations will help elucidate the molecular basis of viral pathogenesis.

### Innate immune effectors

Activation of innate immune pathways is the first line of defense against viral infection in both mammalian and mosquito hosts, but the signaling cascades and effector molecules involved may vary. In mammalian cells, flaviviral infection triggers rapid interferon-centered responses through activation of pattern recognition receptors (PRRs) such as the Toll-like receptors (TLRs), DExD/H-box helicases (RIG-I and MDA5), and protein kinase R (PKR) by viral double-stranded RNA (dsRNA) (100). Viral stress can also activate cytosolic DNA-sensing PRRs, such as the cGAS receptors, by triggering the release of mitochondrial DNA (mtDNA) (101). Activation of these PRRs initiates signaling cascades that result in the production of type I IFNs (IFN-α/β), NF-κB, proinflammatory cytokines, and chemokines, establishing an antiviral state in infected and neighboring uninfected cells. Interferons are the central antiviral cytokines produced by infected mammalian cells. They signal through the JAK-STAT pathway to induce the expression of interferon-stimulated genes, which limit viral infection but also contribute to cellular stress responses, including apoptosis and inflammation, ultimately resulting in tissue damage and disease pathology (102–104). Some PRRs, such as the TLRs and cGAS-like receptors, are described in both human and mosquito cells and have a conserved functional capacity to recognize viral dsRNA and activate downstream immune signaling (105). However, the IFN system and related PRRs, including RIG-I, MDA5, and PKR, are unique to vertebrate hosts (106). In insects, a simplified JAK-STAT pathway exists and is activated by cytokine-like ligands such as Unpaired and Vago, which functionally mimic IFN. This leads to induction of several antiviral effectors, including STAT-responsive genes such as virus-induced RNA 1, defensins, antimicrobial peptides, and complement-like proteins, which limit viral infection but generally do not trigger apoptosis (107–109).

In mosquito cells, the primary PRR is Dicer, a DExD/H-box helicase that recognizes and cleaves viral dsRNA into siRNAs. Viral siRNAs induce RISC-mediated degradation of the viral genome, thus suppressing infection (110, 111). Unlike IFN responses, RNAi-mediated inhibition of viral replication does not result in cell death but reduces viral loads below the threshold required to elicit cytopathic effects (112). Consistent with this, loss-of-function mutations in Dicer-2 have been shown to enhance early replication of several arboviruses, including DENV and YFV, resulting in higher viral loads and increased mortality in infected mosquitoes (113, 114). Despite robust RNAi responses, flaviviruses are not completely cleared from infected mosquito cells. This could be due to viral-encoded suppressors of RNAi (VSRs), such as the DENV NS2A protein and DENV 3′ UTR-derived sub-genomic RNAs (sfRNAs), which dampen RNAi activity and likely allow for persistent, low-level viral replication (115). While RNAi also has antiviral roles in mammalian cells, it is not very robust and is observed only when VSR activity is disrupted. This may arise from intrinsic differences in the mammalian RNAi machinery or from crosstalk of the RNAi pathway with other immune signaling pathways in mammalian cells. For instance, it has been shown that the RNAi pathway can display antiviral functions in pluripotent or undifferentiated mammalian cells where the IFN response is attenuated, but becomes largely inactive in differentiated cells where the IFN response is active (116). Furthermore, laboratory of genetics and physiology 2, which is a RIG-I-like receptor, can compete with Dicer for binding to viral dsRNA substrates in mammalian cells and thus inhibit RNAi activity (117).

Beyond RNAi, mosquitoes possess another unique small RNA-based defense mechanism driven by piwi-interacting RNAs (piRNAs). Unlike virus-derived siRNAs, which are byproducts of viral RNA degradation, viral piRNAs mainly arise from viral sequences that are integrated into the host genome. In infected cells, viral genomic RNA is reverse-transcribed into DNA and integrated into piRNA loci. These integrated viral sequences, known as endogenous viral elements (EVEs), are transcribed to produce viral piRNAs that associate with PIWI proteins to target the complementary viral genomic RNA for degradation (118). Importantly, they also serve as a form of heritable antiviral immune memory that can be transferred across generations. Knockdown of Piwi proteins, including PIWI4 and PIWI5, results in elevated DENV and ZIKV titers in infected mosquito cells (119–122). Furthermore, mosquitoes treated with the reverse transcriptase inhibitor azidothymidine fail to generate EVEs and piRNAs, and consequently succumb to viral infection, whereas untreated controls survive for a much longer period (123, 124). These mosquitoes also produce lower amounts of viral siRNAs, indicating possible crosstalk between the siRNA and piRNA pathways. Ultimately, this results in higher susceptibility of mosquitoes to viral infection, thereby linking these pathways to host survival and viral persistence. In most organisms, including humans and flies, the piRNA pathway is largely confined to germline cells, where it safeguards genomic integrity by silencing transposable elements. In contrast, mosquitoes express an expanded repertoire of Piwi proteins in both germline and somatic tissues, supporting piRNA biogenesis from a broad range of substrates beyond transposons, including protein-coding genes, satellite repeats, and viral RNA, thereby extending its role to antiviral defense (125). Studies have also shown that some mosquito species have a higher capacity to generate piRNA and have a larger repertoire of EVEs, which may influence vector competence (126). Together, the RNAi and piRNA pathways perform specialized functions in mosquitoes and may be directly linked to their ability to support persistent viral infection.

In addition to RNAi and piRNA pathways, the Toll and Imd pathways, which were traditionally associated with antifungal and antibacterial responses, are also activated in response to flaviviral infection in insect cells (106, 127). The Toll pathway is activated by DENV infection, as well as poly I:C treatment, indicating that the insect Toll receptors can function as viral dsRNA sensors, similar to what is observed in mammalian cells. The *Aedes aegypti* Toll6 (AaToll6) was recently shown to share key residues involved in dsRNA binding regions with human TLR3, a well-characterized dsRNA sensor in mammals (128). The Imd pathway shares some similarities with the human TNFR and TLR pathways but has no direct homolog in vertebrates. Imd pathway genes are upregulated in response to DENV infection, but unlike in bacterial infection, where Imd is directly triggered by bacterial peptidoglycan, viral activation of the IMD pathway is indirect, with Dicer-2-mediated RNA sensing and other cellular stress signals acting as major upstream triggers (129). Knockdown of Imd pathway genes in DENV-refractory strains of *Aedes aegypti* results in elevated midgut viral titers, indicating that they are important determinants of host susceptibility to flaviviral infection (130). While the Toll pathway is conserved across humans and mosquitoes, it is hypothesized that the evolution of the IFN system and adaptive immunity in vertebrates may have rendered the Imd pathway redundant, leading to its eventual loss in these lineages. Activation of these pathways leads to nuclear translocation of NF-κB-like transcription factors, Rel1 and Rel2, and subsequent production of a broad repertoire of immune effectors, including AMPs, lysozymes, hemolysins, transferrins, lectins, and complement-like factors (106). The diversity of immune effectors is much greater in invertebrates compared to vertebrates, since the latter can also resort to adaptive immune responses for protection. Other systemic and humoral immune pathways, such as the prophenoloxidase and complement pathways (131, 132), also play important roles in shaping host-specific responses to viral infection but are beyond the scope of this review.

### Stress responses and apoptosis

Upon DENV infection, the rapid accumulation of viral RNA and proteins at the ER activates the integrated stress response (ISR) and unfolded protein response (UPR) pathways, which significantly alter the translational landscape of host cells (133, 134). Stress-induced ER kinases phosphorylate eIF2α, resulting in global inhibition of protein synthesis while selectively promoting translation of stress-responsive mRNAs that help restore cellular homeostasis. PKR is the major ER kinase responsible for translational shutdown in mammalian cells (45), and prolonged ER stress, UPR activation, and translation shutdown trigger apoptosis in infected cells (135–137). Though mosquito cells lack PKR, DENV infection of C6/36 cells has been shown to activate another kinase, PERK, which can phosphorylate eIF2α and block translation initiation (47). It should be noted that C6/36 cells lack a functional RNAi pathway and support abnormally high levels of viral replication (138), so may not be a relevant model for studying infection-induced stress responses. Recent studies in immune-competent mosquito cells have revealed no major changes in eIF2α phosphorylation or global mRNA translation during ZIKV or CHIKV infection. Viral protein synthesis, though, is progressively inhibited over time, which allows cells to survive and supports the establishment of long-term persistent infections (48). These findings suggest that, in addition to RNAi-mediated immune responses, the mechanisms that balance host and viral RNA translation differ significantly in mammalian and mosquito cells and could be a major determinant of infection outcomes in the two hosts.

The remarkable tolerance of mosquito cells to virus-induced ER stress and apoptosis can also be attributed to strong antioxidant and anti-apoptotic responses in these cells (139–141). For instance, DENV infection induces upregulation of several antioxidant genes, including superoxide dismutase, catalase, glutathione peroxidase, glutathione S-transferase (GST), glutaredoxin, thioredoxin, and protein disulfide isomerase in mosquito cells, which neutralize reactive oxygen species (ROS) and preserve redox balance, preventing apoptosis (142–144). Additionally, expression of inhibitor of apoptosis proteins (IAPs) is significantly increased in infected mosquito cells. Combined knockdown of GST and IAP enhances caspase activation and cell death, highlighting the importance of these pathways in maintaining cell survival (145). Interestingly, IAP was upregulated only in DENV-infected mosquito cells, but not in mammalian cells, where a high proportion of cell death was observed through caspase-mediated apoptosis, highlighting a key difference in host responses to DENV. DENV infection also upregulates the p53-2 isoform, which reduces ROS levels via induction of catalase (143, 146). Together, these host-specific protective mechanisms likely allow mosquito cells to sustain viral replication without undergoing apoptosis.

ER stress also activates autophagy as a compensatory mechanism to restore cellular homeostasis during infection. This process is frequently exploited by many viruses to enhance viral replication (135). In DENV-infected mammalian cells, viral NS4A and NS1 proteins directly induce autophagosome formation and promote viral replication in multiple ways, including release of metabolites required for the formation and function of vROs and the remodeling of intracellular membranes (133, 147–150). Inhibition of autophagy using chemical inhibitors or gene silencing significantly reduces DENV replication and viral titers. In mosquitoes, the role of autophagy is more context-dependent. Although the core autophagy machinery is conserved, insects possess single isoforms of most autophagy-related genes, limiting functional redundancy (151, 152). DENV infection can induce the expression of autophagy-related genes in mosquito midguts and in C6/36 cells, but the effects of autophagy induction on viral infection vary with cell type and experimental conditions. In Aag2 cells, chemical induction of autophagy using rapamycin resulted in reduced DENV titers, suggesting an antiviral role (153). However, knockdown of core autophagy genes did not alter DENV-2 titers. Similarly, in C6/36 cells, autophagy modulators had minimal effect on DENV-2 replication (154). Thus, there is no consistent evidence on the role of the autophagy pathway in modulating DENV infection in mosquito hosts.

**Fig 2:**
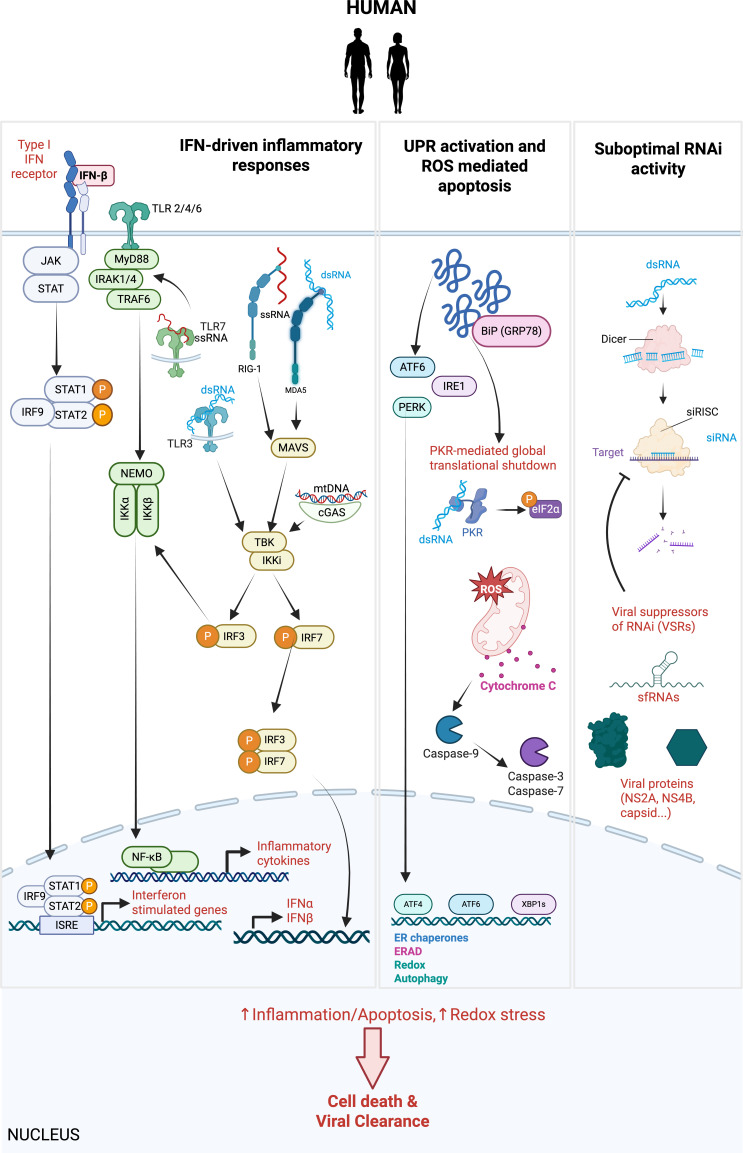
Antiviral and cellular stress responses to flavivirus infection in mammalian cells. Flavivirus infection in mammalian cells triggers multiple innate immune and cellular stress pathways that collectively promote viral restriction and clearance. Viral RNA is detected by TLRs, RIG-I-like receptors, and cGAS-associated signaling pathways, leading to activation of interferon signaling and production of inflammatory cytokines. Infection also induces ER stress, ROS accumulation, apoptosis, and PKR-mediated translational shutdown, all of which contribute to limiting viral replication. Although RNAi pathways are present, their antiviral role is generally less prominent than interferon-mediated defenses. (Created in BioRender [S. Shivaprasad, 2026, https://BioRender.com/josderp].)

**Fig 3:**
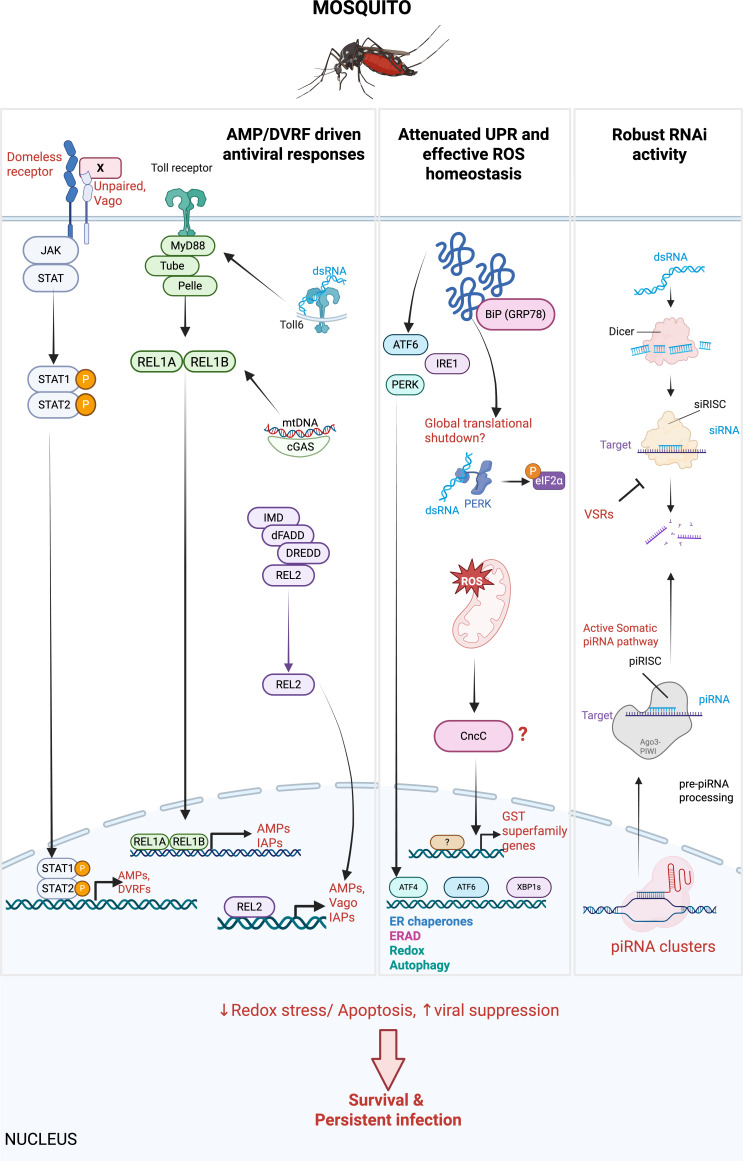
Antiviral responses and stress tolerance mechanisms in flavivirus-infected mosquito cells. Mosquito cells employ distinct antiviral and stress response mechanisms that control viral infection and support viral persistence. In the absence of canonical interferon signaling, antiviral defense relies primarily on the Toll, IMD, JAK-STAT, RNAi, and piRNA pathways. Dicer-2- and Piwi-mediated small RNA responses play central roles in suppressing viral replication. Compared with mammalian cells, mosquito cells exhibit attenuated ER stress and ROS-induced apoptosis and enhanced redox homeostasis, which promote viral tolerance and facilitate long-term persistent infection without substantial cellular damage. (Created in BioRender [S. Shivaprasad, 2026, https://BioRender.com/josderp].)

Overall, intrinsic differences in stress responses, including ER stress adaptation, antioxidant capacity, and anti-apoptotic defenses, contribute to the divergent outcomes of DENV infection in mammalian and mosquito hosts (Fig. 2 and 3). In mammalian cells, resolution of infection depends on maintaining a balance between viral clearance and restoration of cellular homeostasis. However, prolonged ER and mitochondrial stress, metabolic dysfunction, and sustained IFN signaling can overwhelm these adaptive responses, ultimately resulting in global translational shutdown, apoptosis, and pathological inflammation. In contrast, mosquito cells appear to be better adapted to tolerate persistent viral infection. ER stress pathways are not robustly activated, and mitochondrial stress is more effectively controlled due to strong antioxidant defenses. In addition, RNAi pathways help restrict viral replication and maintain viral loads below pathogenic thresholds. Metabolic pathways also contribute to host tolerance mechanisms. For example, sugar feeding promotes the expression of antiviral genes such as Ago2 and Piwi4 in the intestinal tract of *Aedes aegypti* and protects mosquitoes from arboviral infections, likely through effects on central carbon metabolism (154). Similarly, trehalose, the primary circulating sugar in insects, not only primes immune responses but also functions as an antioxidant and regulator of autophagy (155). Together, these adaptations may enable mosquitoes to support persistent flaviviral infection while minimizing cellular damage and inflammatory pathology. Further studies investigating the interplay between stress responses, innate immune pathways, metabolic reprogramming, and viral evasion strategies will provide important insights into the determinants of infection outcomes and the mechanisms underlying viral persistence. Some of the outstanding questions in the field are highlighted in Box 1.

Box 1. **Open questions**

**What are the molecular determinants of host tropism and intracellular compatibility across mosquito and mammalian hosts?**

How do differences in receptor usage, membrane composition, and host factor availability shape viral replication kinetics, species compatibility, and vector competence across hosts?

**What governs the balance between viral replication and host cell survival, particularly in mosquito cells, which support persistent infection?**

How do mosquito cells sustain long-term viral replication without cytopathic effects? How do innate immune, translational, and stress responses, and antioxidant and anti-apoptotic defenses collectively establish and maintain an equilibrium between productive viral replication and host tolerance?

**How do immune and metabolic constraints intersect to shape viral adaptation strategies in divergent hosts?**

What are the RNAi-dependent and RNAi-independent mechanisms that influence viral replication and persistence across hosts? How do metabolic-immune interfaces differ fundamentally between acute and persistent infection models?

**What defines the evolutionary boundaries of host range expansion and constrains host-virus coevolution?**

Which specific intracellular barriers impose the strongest evolutionary hurdles for cross-species transmission? What molecular signatures can predict a virus's potential for host range expansion?

### Host intracellular environments in viral emergence

In the sections above, we have discussed how host intracellular environments shape viral infection efficiencies and outcomes across humans and mosquitoes, and even across different mosquito species. While the primary focus of this review has been on comparing the molecular determinants of flaviviral infection across human and mosquito hosts, the extracellular environment plays an equally important role in influencing host tropism and vector competence. Factors such as temperature, pH, microbiota—particularly *Wolbachia*, the extracellular matrix, secreted proteases, and humoral and adaptive immune responses across hosts all contribute to viral infection, transmission, and host specificity, but are beyond the scope of this review.

The intrinsic evolvability of viruses, driven by large population sizes, short generation times, and high mutation rates, continuously generates diverse viral variants. However, both intracellular and extracellular host environments impose strong selection pressures at every stage of the viral life cycle, determining which variants replicate efficiently and persist. At the level of viral entry, the repertoire of surface receptors expressed by host cells determines the selection of viral envelope protein variants that show strong receptor binding. After entry, the availability of host factors required for different stages of the viral life cycle, the activity of innate immune sensors that recognize viral RNA, the host metabolic state, and cellular stress response and tolerance pathways collectively define which viral variants perform well within a given host. Since flaviviruses must alternate between two phylogenetically distant hosts, only those variants that can tolerate or adapt to both environments are maintained through transmission cycles. This imposes fundamental evolutionary constraints on flaviviruses and favors the selection of generalist viruses with broad compatibility rather than single host-specialized variants. To enhance replication in specific hosts, flaviviruses often adopt modular adaptive strategies, wherein distinct regions of the viral RNA or proteins are selectively optimized for host-specific functions while preserving their compatibility in the alternate host. A well-characterized example is the flaviviral 3′ UTR, which contains duplicated structural elements that accumulate distinct mutations in human and mosquito cells, resulting in the production of different sfRNA isoforms (156). Studies with DENV have shown that host-specific selection pressures favor the generation of shorter sfRNAs in mosquitoes but longer sfRNAs in mammalian cells, enabling antagonism of different antiviral pathways in the two hosts with minimal impact on viral fitness.

Identifying intracellular constraints to cross-species transmission of viruses is critical for predicting which variants are most likely to emerge. Unlike ecological or epidemiological parameters, these barriers can be directly assessed by adapting viruses to different host cells in culture. Insect-specific flaviviruses (ISFs) provide a useful model system for identifying barriers that limit host range, as they are closely related to mosquito-borne flaviviruses but replicate only in mosquito hosts. Zhang et al. showed that while ISFs can enter mammalian cells, their UTRs fail to recruit host factors required for efficient replication. Replacing ISF UTRs with those from Zika virus, which replicates in both insect and human hosts, restores replication, as ZIKV UTRs can recruit host factors such as G3BP1, SYNCRIP, and WTAP that are essential for replication in mammalian cells (157). These findings highlight that host-specific intracellular factors act as stringent filters on viral genomes, and only variants that acquire the ability to effectively engage these pathways can expand their host range. Insect-specific flaviviruses that demonstrate partial intracellular compatibility, such as limited replication in human cells or partial resistance to interferon, represent high-priority surveillance targets, as they may require only minor genetic changes to achieve full human adaptation.

Thus, viral emergence is ultimately driven by the selection of variants that overcome intracellular barriers and achieve functional compatibility across hosts. Identifying the specific molecular and cellular constraints that limit replication in each host will be critical for predicting which adaptive mutations are likely to be selected during evolution and which viruses have the potential to cross species barriers. Ultimately, a mechanistic understanding of these host-imposed barriers will be essential for anticipating future outbreaks and developing targeted strategies to disrupt transmission cycles before widespread emergence occurs.

## SUMMARY

The intracellular environment and signaling pathways are a key determinant of viral infection efficiencies and outcomes. Most of the host factors co-opted by DENV and other flaviviruses across both human and mosquito cells are highly conserved due to the overall similarities in the viral life cycle and include conserved functional modules such as ER membranes, chaperone complexes, and lipid metabolic pathways. Some species-specific differences in host factor usage have been observed but need more rigorous analyses. The divergent outcomes of flaviviral infection in mammalian and mosquito hosts arise from fundamental differences in how each host balances stress tolerance and immune activation. In mammalian cells, rapid viral replication overwhelms ER homeostasis, triggering strong interferon responses and apoptosis that restrict infection but contribute to cytopathic effects and disease. In contrast, mosquito cells maintain a more balanced equilibrium where viral replication is slower and buffered by strong antioxidant and anti-apoptotic defenses and immune responses such as the RNAi and piRNA pathways that limit viral replication, supporting long-term persistence and transmission. These physiological differences parallel those observed in other reservoir–spillover systems, such as the tolerance of bats to persistent infection with coronaviruses and Nipah virus (158, 159). Even between mosquito strains, variations in host factor availability, immune signaling, and metabolic state influence vector competence and susceptibility. Understanding how flaviviruses adapt to diverse cellular environments while navigating host-specific constraints will be critical for elucidating the molecular basis of replication, persistence, host adaptation, and vector competence.
